# Tap water microbiome shifts in secondary water supply for high-rise buildings

**DOI:** 10.1016/j.ese.2024.100413

**Published:** 2024-03-16

**Authors:** Manjie Li, Zhaowei Liu, Yongcan Chen

**Affiliations:** a Shenzhen International Graduate School, Tsinghua University, Shenzhen, 518055, PR China; b State Key Laboratory of Hydroscience and Engineering, Tsinghua University, Beijing, 100084, PR China

**Keywords:** Secondary water supply systems (SWSSs), Tap water, Bacterial community, Water chemistry

## Abstract

In high-rise buildings, secondary water supply systems (SWSSs) are pivotal yet provide a conducive milieu for microbial proliferation due to intermittent flow, low disinfectant residual, and high specific pipe-surface area, raising concerns about tap water quality deterioration. Despite their ubiquity, a comprehensive understanding of bacterial community dynamics within SWSSs remains elusive. Here we show how intrinsic SWSS variables critically shape the tap water microbiome at distal ends. In an office setting, distinct from residential complexes, the diversity in piping materials instigates a noticeable bacterial community shift, exemplified by a transition from α-Proteobacteria to γ-Proteobacteria dominance, alongside an upsurge in bacterial diversity and microbial propagation potential. Extended water retention within SWSSs invariably escalates microbial regrowth propensities and modulates bacterial consortia, yet secondary disinfection emerges as a robust strategy for preserving water quality integrity. Additionally, the regularity of water usage modulates proximal flow dynamics, thereby influencing tap water's microbial landscape. Insights garnered from this investigation lay the groundwork for devising effective interventions aimed at safeguarding microbiological standards at the consumer's endpoint.

## Highlights

• Secondary water supply systems critically shape tap water microbiome at distal ends.
• Plumbing materials determine the dominant taxon and pathogens regrowth potential.
• Secondary disinfection is necessary to protect microbiological water quality.
• Hydraulic retention time of 6–12 h is conducive to water quality stability.
• Shifts in bacterial community are associated with the changes of water chemistry.

## Graphical abstract

**Figure:**
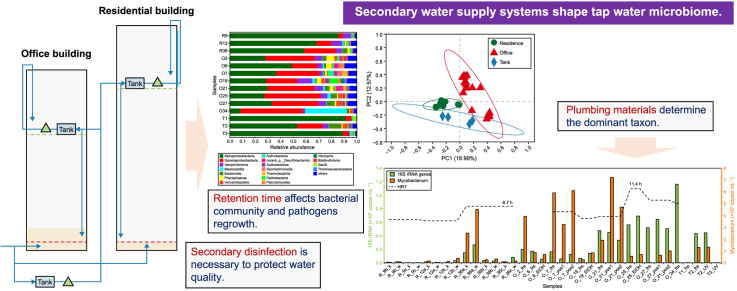

## 1 Introduction

The presence and regrowth of microbes are inevitable in drinking water distribution systems despite disinfection treatment, posing threats to microbes-related problems, including nitrification [1], bio-corrosion [2], and the persistence of pathogens [[3], [4], [5]]. In addition to the origin from source water and treatment process, bacteria can be released from the biofilms and deposits developed on the pipe surface into bulk water [6,7], leading to the deterioration of microbiological water quality and shifts in the bacterial community at consumers’ taps.

Secondary water supply has become the primary method for high-rise buildings in metropolitan cities [[8], [9], [10]]. Similar to premise plumbing, secondary water supply systems (SWSSs) are the portion of a water supply system connected to the main distribution system via service lines and directly serve the consumers. Accordingly, overnight stagnation of water [11,12] and presence of opportunistic pathogens [[3], [4], [5]] can be anticipated in SWSSs, which have been determined in premise plumbing systems.

**Fig. 1:**
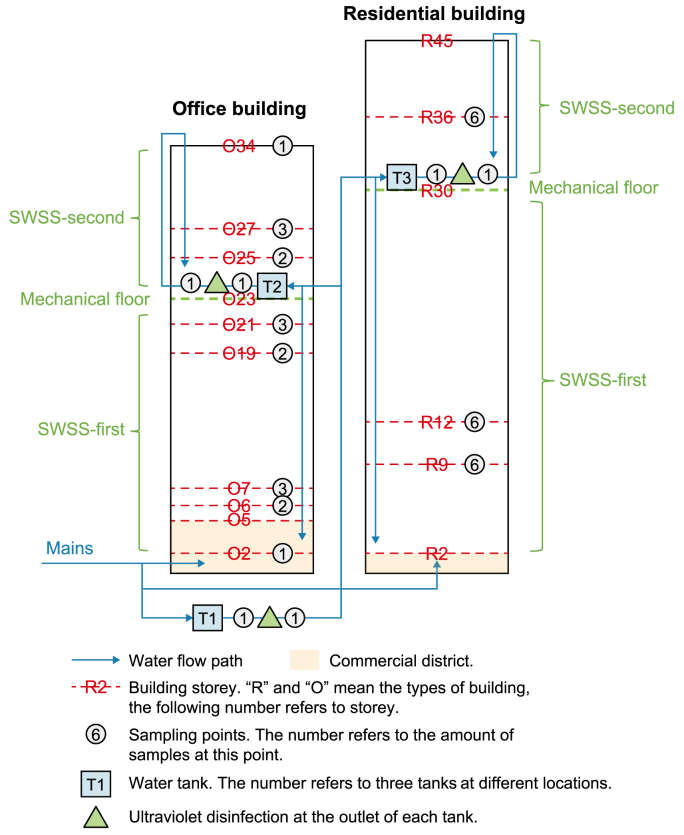
Diagram of the study area and sampling scheme. Sampling was conducted in an architectural complex consisting of residential and office buildings. For both buildings, consumers on the 1st and 2nd floors are directly supplied by the mains, and the rest above are served by secondary water supply systems (SWSSs). In SWSSs, treated water is transported via the mains to an underground tank “T1”, disinfected using ultraviolet, and pumped to the mechanical floors. A part of the water flows by gravity to serve the consumers below the mechanical floor, marked with “SWSS-first”. The rest is stored in a tank, followed by ultraviolet-disinfected, and pumped to serve the consumers above the mechanical floor, marked with “SWSS-second”. Samples were collected from the 9th, 12th, and 36th floors of the residential building, the 2nd, 6th, 7th, 19th, 21st, 25th, 27th, and 34th floors of the office building, and the outlets of the three tanks, with the number of samples marked in the diagram.

The primary difference from premise plumbing is that SWSSs are usually applied in high-rise buildings (with a storey number larger than five), which include water tanks, secondary disinfection facilities, pumps, and more complex pipelines to store, pressurize, and transport water to consumers’ taps (Fig. 1) [13]. Consequently, SWSSs show a variable flow condition, long water retention time, low disinfectant residual, high specific pipe surface area, and warm temperature [9,10], providing a favorable environment for microbial regrowth involved with large uncertainties [14]. The complex topology and various household water consumption [15,16] lead to complicated flow characteristics within SWSSs, thus significantly influencing the transfer of solutes [17,18] and the transport of microorganisms [19] in bulk water, which is difficult to identify. Besides, water quality is only monitored in main distribution pipes but not within SWSSs by water regulations. Unified administration of SWSSs is challenging as in-building infrastructures are usually managed individually, which may lack professionalism, rather than by water companies. SWSS design and maintenance optimization is indispensable to protect the microbiological water quality in high-rise buildings.

The contributions of source water [7,[20], [21], [22]], treatment process [7,23,24], and distribution systems [7,25] to the microbiome in tap water have been determined and quantified. However, the potential influence of the household pipes and premise plumbing system on tap water microbiota is not fully understood. Particularly, water quality deterioration in SWSSs is always neglected, directly impacting public health. Chan et al. [8] combined field sampling and computational modeling to unravel the water metallic quality in SWSSs. Geng et al. [9] developed a water age and chlorine decay model in SWSSs, which significantly correlated with microbial regrowth in drinking water. Li et al. [10] performed tap water sampling in more than 20 high-rise buildings with various types of SWSSs, tank materials, water sources, storey numbers, and building ages, highlighting the critical role of SWSSs in shaping the drinking water microbiota. While the inter-SWSSs differences have been carefully considered, the intra-SWSSs variations and their influencing factors are completely dark. Determining the vulnerable areas for microbiological water quality within SWSSs is essential. Furthermore, the significant inter-SWSSs differences and temporal variations always mask the influence of intra-SWSSs design factors. A profile of bacterial community shifts during secondary water supply is imperative via a thorough investigation.

This study develops a comprehensive sampling protocol to determine water microbiota shifts within SWSSs. Subsequently, the hydraulic retention time (HRT) was estimated to reflect the effect of water flow. To thoroughly investigate the intra-SWSSs influencing factors, an architectural complex consisting of different functional areas and various water supply strategies is selected for experiments. The results of this study can provide important insights into how and to what extent the characteristics of SWSSs would affect the bacterial community in tap water. Based on the findings, practical technologies can be developed to protect the microbiological water quality at consumers’ taps.

## 2 Materials and methods

### 2.1 Site location and sample collection

The study was conducted in an architectural complex built in 2018, consisting of residential areas, office buildings, and commercial districts in Beijing, China. As shown in Fig. 1, tap water was sampled from a residential building (total number of storeys: 45) and an office building (total number of storeys: 34). For the residential building, the first two floors consist of small shops and the above floors are used for residences, with three apartments for each floor. The office building contains a shopping mall on the 1st–5th floors, with the remaining floors dedicated to office space. Each office floor comprises roughly ten office rooms and one shared washroom. The residential building is equipped with brass taps, whereas the office building uses stainless steel taps. Commercial districts were not included in the present study due to the non-operational status of the shopping mall and most shops at the time of sampling.

The mains directly supply consumers on the 1st and 2nd floors for both buildings. The rest above are served by SWSSs (Fig. 1). In SWSSs, treated water is transported via the mains to an underground tank, “Tank1”, made of stainless steel, disinfected using ultraviolet, and pumped to the mechanical floors (the 23rd floor for the office building and the 30th floor for the residential building). A part of the water flows by gravity to serve the consumers below the mechanical floor (except the 1st and 2nd floors), marked with “SWSS-first”. The rest is stored in a tank (“Tank2” for the office building and “Tank3” for the residential building), ultraviolet-disinfected again, and pumped to serve the consumers above the mechanical floor, marked with “SWSS-second”. Generally, intra-building horizontal pipes in the office building are made of galvanized plastic-lined steel (GPLS) with a 25–30 mm diameter. In contrast, polypropylene pipes are used in the residential building. GPLS (<50 mm) and cast iron pipes (≥50 mm) are used for standpipes and connections to mains.

The survey was designed to include different building functional areas, storeys, water supply strategies, tap locations, frequency of tap utilization, and influence of tap disinfection and post-flushing. The “first-draw” samples (2 L water before flushing the taps) were collected after overnight stagnation, which has been considered the worst-case scenario of contaminant release and bacterial regrowth [8,11,26]. For the residential building, sampling was conducted on the 9th, 12th, and 36th floors (Fig. 1) by carefully considering the above factors and residents’ willingness. The residents were asked to confirm that they had no pandemic-related lockdown during the one month before this sampling campaign. The taps were carefully checked to ensure the absence of an extra filter. Both the kitchen tap water and washroom tap water were sampled and marked with “k” and “w”, respectively, from all three apartments of each selected floor (Table S1). The residents were given sterile jars in advance and trained to collect 2-L “first-draw” samples in the morning according to the instructions. For the office building, the “first-draw” sample was taken from the washroom taps on eight selected floors, namely the 2nd (the lobby of the office building), 6th, 7th, 19th, 21st, 25th, 27th, and 34th floors, to cover different storeys and water consumption levels, marked with “fre”. In addition, an extra sample was collected after tap disinfection with 75% ethanol and 3-min flushing from the 6th, 19th, and 25th floors, marked with “EtOH”. On the 7th, 21st, and 27th floors, two extra post-flushing samples were taken after 10- and 18-L flushing and marked with “pos1” and “pos2”, respectively. Water was also sampled from the underground tank and two tanks on the mechanical floors (Fig. 1). Samples before and after disinfection were taken via the taps equipped at the inlet and outlet of the ultraviolet disinfection facilities, marked with “fre” and “UV”, respectively. To minimize the temporal effects, the whole sampling was performed simultaneously in the early morning (6:30–8:30 a.m.) of a weekday in July 2021. Due to a communication or accessibility problem, five samples were not successfully collected (Table S1). The remaining samples were transported to the laboratory with ice within 2 h. Table S1 shows detailed information on the samples. Data on water consumption in summer were given to represent the frequency of tap utilization.

### 2.2 Water quality analysis

Water temperature and chlorine residual were measured in situ using an electrochemistry tester (YSI Proquatro, USA) and a HACH DR300 instrument (USA). The water temperature is ∼24 °C for all samples. Chlorine residual was verified negligible due to using ultraviolet as secondary disinfection. Other parameters, including water pH, turbidity, chloride, fluoride, sulfate, total dissolved solids (TDS), hardness, chemical oxygen demand (COD), ammonia nitrogen, nitrite nitrogen, heterotrophic plate count (HPC), total coliforms, and heavy metals, were determined in the laboratory using 0.5-L of the water sample according to the standard methods [27].

### 2.3 DNA extraction and Illumina sequencing

The remaining 1.5 L of water was filtered through a 0.22 μm sterile filter. Genomic DNA was extracted using an Invitrogen kit (Thermo Fisher Scientific, USA) according to the manufacturer's instructions. Then, its quality and concentration were determined by 1% agarose gel electrophoresis and kept at −80 °C before further use. The hypervariable region V3–V4 of the bacterial 16S rRNA gene was amplified with primer pairs 338F (5′-ACTCCTACGGGAGGCAGCAG-3′) and 806R (5′-GGACTACHVGGGTWTCTAAT-3′) using an ABI GeneAmp® 9700 thermocycler (ABI, USA). The barcode information is provided in Table S2. The polymerase chain reaction (PCR) mixture included 4 μL 5 × Fast Pfu buffer, 2 μL 2.5 mM dNTPs, 0.8 μL each primer (5 μM), 0.4 μL Fast Pfu polymerase, 0.2 μL BSA, 10 ng template DNA, and ddH_2_O to a final volume of 20 μL. The PCR amplification cycling conditions were as follows: initial denaturation at 95 °C for 3 min, followed by 27 cycles of denaturing at 95 °C for 30 s, annealing at 55 °C for 30 s and extension at 72 °C for 45 s, single extension at 72 °C for 10 min, and end at 10 °C. Each sample was amplified in triplicates. The PCR products were extracted from 2% agarose gel, purified using the AxyPrep DNA Gel Extraction Kit (AxyGen Biosciences, USA), and quantified using a QuantiFluor™-ST fluorometer (Promega, USA). Purified amplicons were pooled in equimolar amounts and paired-end sequenced on an Illumina MiSeq PE300 platform (Illumina, USA) by the Majorbio Bio-Pharm Technology Co., Ltd (China). The raw sequencing reads were deposited into the NCBI Sequence Read Archive (SRA) database (Accession Number: PRJNA886672).

### 2.4 Quantitative polymerase chain reaction (qPCR)

16S rRNA genes were enumerated by quantitative polymerase chain reaction (qPCR) to indicate the magnitude of total bacteria in drinking water [1,10,28]. It was performed with primer pairs 1368F (5′-CGGTGAATACGTTCYCGG-3′) and 1492R (5′-CGWTACCTTGTTACGACTT-3′) on an ABI7300 real-time PCR system (Applied Biosystems, USA) according to previously established methods [10]. The PCR mixture included 10 μL 2 × Taq Plus Master Mix, 0.8 μL each primer (5 μM), 1 μL template DNA, and 7.4 μL ddH_2_O. The PCR amplification cycling conditions were as follows: initial denaturation at 95 °C for 5 min, followed by 35 cycles of denaturing at 95 °C for 30 s, annealing at 55 °C for 30 s, and extension at 72 °C for 1 min. Each qPCR run was performed in triplicates. The standard curve was generated using a ten-fold serial dilution of standard DNA. The PCR amplification efficiency was 100.51%, with the correlation coefficient (*R*^2^) greater than 0.99.

Targeted genes for *Mycobacterium* spp., *Legionella* spp., *Legionella pneumophila*, *Mycobacterium avium*, *Acanthamoeba* spp., and *Vermamoeba vermiformis*, which have been recognized as opportunistic pathogens in drinking water distribution systems, were also enumerated by qPCR using previously established methods (Table S3) [10]. However, most of them failed to amplification in the preliminary experiment, and thus, only *Mycobacterium* spp. was processed in the subsequent analysis.

### 2.5 Sequence processing and statistical analysis

Due to failed DNA amplification, sample “T1_UV” was excluded (Table S1). The sequences of the remaining samples were filtered with fastp (v0.19.6) and merged with FLASH (v1.2.7). Then, the high-quality sequences were de-noised using DADA2, which generated amplicon sequence variants (ASVs). Taxonomic assignment of ASVs was performed using the Naive Bayes consensus taxonomy classifier in Qiime2 and the SILVA 16S rRNA database (v138). The analysis of variance (ANOVA) was performed following the Shapiro-Wilk normality test to determine the significance of differences in water quality and α-diversity indices. The principal coordinate analysis (PCoA) using Bray-Curtis distance was performed to demonstrate the β-diversity of samples, and the clustering was further evaluated using the analysis of similarity (ANOSIM). Canonical correspondence analysis (CCA) and Pearson correlation analysis were conducted to reveal the correlations between bacterial community profiles and water physicochemical parameters.

### 2.6 Estimation of HRT

The HRT was estimated to reflect the flow and transportation processes within the SWSSs. The HRT in Tank1, Tank2, and Tank3 (Fig. 1) was labeled as $T_{\text{tank}1}$, $T_{\text{tank}2}$, and $T_{\text{tank}3}$, respectively, which is usually regulated by the building managers to avoid prolonged storage. The volumes of single-pass standpipe ($V_{s}$) and horizontal supply line ($V_{h}$) for each floor can be estimated by equations (1), (2), respectively:

(1) $$V_{s}=\frac{1}{4}\pi {d_{s}}^{2}h$$

(2) $$V_{h}=\frac{1}{4}\pi {d_{h}}^{2}l$$

where $d_{s}$ and h are the diameter and height of the standpipe for each floor, m, and $d_{h}$ and l are the diameter and length of the horizontal supply line on each floor, m.

As Tank1 is located on the underground 2nd floor, water travelling time from Tank1 to the mechanical floor in the office building (Fig. 1) can be calculated as $\frac{\left(23+2\right)V_{s}}{\sum_{i=6}^{34}Q_{O_{i}}}$, and that from Tank2 to the 34th floor can be calculated as $\frac{\left(34-23\right)V_{s}}{\sum_{i=24}^{34}Q_{O_{i}}}$, where $Q_{O_{i}}$ (m^3^ s^−1^) represents the average water consumption rate at the *i*th floor. Considering the water flowing down to SWSS-first area, water travelling time through the *j*th floor can be calculated as $\frac{V_{s}}{\sum_{i=6}^{j}Q_{O_{i}}}$, and thus the total travelling time from the mechanical floor to taps on the *k*th floor can be estimated as $\sum_{j=k}^{23}\frac{V_{s}}{\sum_{i=6}^{j}Q_{O_{i}}}+\frac{V_{h}}{Q_{O_{k}}}$. Similarly, the water travelling time from the 34th floor to SWSS-second area is $\sum_{j=k}^{34}\frac{V_{s}}{\sum_{i=24}^{j}Q_{O_{i}}}+\frac{V_{h}}{Q_{O_{k}}}$. Accordingly, the HRT of tap water within SWSSs in the office building can be calculated by equation (3).

(3) $$\text{HRT}=\{\begin{array}{c} T_{\text{tank}1}+\frac{\left(23+2\right)V_{s}}{\sum_{i=6}^{34}Q_{O_{i}}}+\sum_{j=k}^{23}\frac{V_{s}}{\sum_{i=6}^{j}Q_{O_{i}}}+\frac{V_{h}}{Q_{O_{k}}}\;\left(k=6–23\right)\\ T_{\text{tank}1}+T_{\text{tank}2}+\frac{\left(23+2\right)V_{s}}{\sum_{i=6}^{34}Q_{O_{i}}}+\frac{\left(34-23\right)V_{s}}{\sum_{i=24}^{34}Q_{O_{i}}}+\sum_{j=k}^{34}\frac{V_{s}}{\sum_{i=24}^{j}Q_{O_{i}}}+\frac{V_{h}}{Q_{O_{k}}}\;\left(k=24–34\right) \end{array}$$

Similarly, the HRT of tap water within SWSSs in the residential building can be approximately estimated by equation (4).

(4) $$\text{HRT}=\{\begin{array}{c} T_{\text{tank}1}+\frac{\left(30+2\right)V_{s}}{\sum_{i=3}^{45}Q_{R_{i}}}+\sum_{j=k}^{30}\frac{V_{s}}{\sum_{i=3}^{j}Q_{R_{i}}}+\frac{V_{h}}{Q_{R_{k}}}\;\left(k=3–30\right)\\ T_{\text{tank}1}+T_{\text{tank}3}+\frac{\left(30+2\right)V_{s}}{\sum_{i=3}^{45}Q_{R_{i}}}+\frac{\left(45-30\right)V_{s}}{\sum_{i=31}^{45}Q_{R_{i}}}+\sum_{j=k}^{45}\frac{V_{s}}{\sum_{i=31}^{j}Q_{R_{i}}}+\frac{V_{h}}{Q_{R_{k}}}\;\left(k=31–45\right) \end{array}$$

where $Q_{R_{i}}$ represents the average water consumption rate at the *i*th floor in the residential building, m^3^ s^−1^.

## 3 Results

### 3.1 Water quality characteristics

The quality of all tap water samples conforms to the national standards for drinking water [13]. Table 1 shows the statistics of water quality parameters regarding different sample classification criteria, with the standard guidelines also given for comparison. ANOVA shows no significant difference associated with tap disinfection, tap location, and post-flushing; thus, the relevant statistical results are not given. Data on some parameters, such as turbidity, fluoride, ammonia nitrogen, nitrite nitrogen, total coliforms, Al, Cr, Cd, and Pb, are not given because they are below the respective detection limits. Significant differences in water quality can be determined with different functional areas. Cu content is relatively high in the tap water from residences (0.069 ± 0.076 mg L^−1^, *p* = 0.002 in ANOVA), whereas it is below the detection limit (0.009 mg L^−1^) in all the samples from the office building and water tanks. Samples from the water tanks are characterized by high chloride (14.1 ± 0.7 mg L^−1^) and TDS (165 ± 3 mg L^−1^) levels (*p* < 0.001). Comparing different tanks, samples from Tank1 are more abundant in Fe (0.12 ± 0.01 mg L^−1^), while samples from Tank2 are richer in Zn (0.19 ± 0.01 mg L^−1^, data not shown in Table 1).

**Table 1:** Statistics of water quality parameters regarding different sample-classification criteria.

Classification criteria	Water samples	pH	Chloride	Sulfate	TDS	Hardness	COD	HPC	Fe	Mn^a^	Cu	Zn
			(mg L^−1^)	(mg L^−1^)	(mg L^−1^)	(mg L^−1^ as CaCO_3_)	(mg L^−1^)	(CFU mL^−1^)	(mg L^−1^)	(mg L^−1^)	(mg L^−1^)	(mg L^−1^)
Guidelines^b^		6.5–8.5	250	250	1000	450	3	100	0.3	0.1	1.0	1.0
Functional areas^c^	Residence (*n* = 14)	8.24 ± 0.12	10.5 ± 1.2	19.0 ± 1.7	155 ± 3	90.3 ± 1.4	0.70 ± 0.04	60 ± 22	0.024 ± 0.010	0.0020 ± 0.0050	0.069 ± 0.076∗∗	0.10 ± 0.04
	Office (*n* = 17)	8.16 ± 0.14	10.9 ± 1.1	17.9 ± 1.4	154 ± 7	91.7 ± 1.8	0.69 ± 0.04	46 ± 22	0.040 ± 0.028	0.0048 ± 0.0064	0.005 ± 0.000	0.40 ± 0.24∗∗
	Water tank (*n* = 5)	8.05 ± 0.03	14.1 ± 0.7∗∗	19.2 ± 0.8	165 ± 3∗∗	89.6 ± 1.7	0.74 ± 0.03	51 ± 17	0.062 ± 0.056	0.0003 ± 0.0002	0.007 ± 0.003	0.12 ± 0.07
Water supply (for residence)	SWSS-first (*n* = 8)	8.25 ± 0.13	9.8 ± 0.6∗	18.4 ± 1.8	155 ± 3	90.4 ± 1.4	0.70 ± 0.04	57 ± 23	0.021 ± 0.012	0.0031 ± 0.0065	0.048 ± 0.028	0.10 ± 0.04
	SWSS-second (*n* = 6)	8.22 ± 0.13	11.5 ± 1.0	19.8 ± 1.2	156 ± 1	90.2 ± 1.6	0.70 ± 0.05	64 ± 21	0.026 ± 0.006	0.0005 ± 0.0003	0.097 ± 0.111	0.10 ± 0.05
Water supply (for office)	Mains (*n* = 1)	8.31	11.5	19.0	154	92.5	0.62	98	0.019	0.0003	0.005	0.16
	SWSS-first (*n* = 10)	8.12 ± 0.12	10.9 ± 1.3	17.5 ± 1.2	157 ± 8	91.7 ± 1.8	0.69 ± 0.03	40 ± 15	0.051 ± 0.032	0.0060 ± 0.0080	0.005 ± 0.000	0.44 ± 0.30
	SWSS-second (*n* = 6)	8.21 ± 0.15	10.9 ± 0.6	18.3 ± 1.6	149 ± 2	91.5 ± 2.0	0.69 ± 0.05	49 ± 21	0.026 ± 0.009	0.0035 ± 0.0024	0.005 ± 0.000	0.39 ± 0.06
Storey (for residence)	9th (*n* = 4)	8.14 ± 0.04	9.7 ± 0.3	17.0 ± 0.8∗∗	153 ± 4	91.0 ± 1.5	0.72 ± 0.03	59 ± 17	0.016 ± 0.006	0.0118 ± 0.0114	0.065 ± 0.029	0.10 ± 0.04
	12th (*n* = 4)	8.36 ± 0.07	9.9 ± 0.9	19.8 ± 1.3	156 ± 2	89.7 ± 1.0	0.69 ± 0.04	56 ± 31	0.027 ± 0.014	0.0050 ± 0.0094	0.030 ± 0.012	0.11 ± 0.04
	36th (*n* = 6)	8.22 ± 0.13	11.5 ± 1.0∗∗	19.8 ± 1.2	156 ± 1	90.2 ± 1.6	0.70 ± 0.05	64 ± 21	0.026 ± 0.006	0.0005 ± 0.0003	0.097 ± 0.111	0.10 ± 0.05
Storey (for office)	2nd–7th (*n* = 6)	8.16 ± 0.14	11.3 ± 1.5	17.7 ± 1.0	155 ± 10	92.1 ± 1.2	0.68 ± 0.05	52 ± 28	0.052 ± 0.043	0.0021 ± 0.0029	0.005 ± 0.000	0.22 ± 0.16∗
	19th–34th (*n* = 11)	8.16 ± 0.14	10.7 ± 0.7	18.0 ± 1.5	153 ± 6	91.5 ± 2.0	0.69 ± 0.04	44 ± 19	0.033 ± 0.014	0.0062 ± 0.0073	0.005 ± 0.000	0.50 ± 0.22
Water consumption (for office)	1–5 t (*n* = 9)	8.13 ± 0.12	10.5 ± 0.7	17.1 ± 1.3∗	153 ± 7	92.3 ± 2.1	0.68 ± 0.35	46 ± 18	0.055 ± 0.031	0.0076 ± 0.0076	0.005 ± 0.000	0.49 ± 0.27
	10–14 t (*n* = 7)	8.18 ± 0.16	11.4 ± 1.4	18.7 ± 1.0	155 ± 9	90.8 ± 1.1	0.71 ± 0.43	40 ± 18	0.024 ± 0.006∗	0.0017 ± 0.0020	0.005 ± 0.000	0.32 ± 0.15

∗ and ∗∗ mean the significant difference at *p* < 0.05 and *p* < 0.01 levels in ANOVA, respectively, compared to any other population within the current sample-classification criterion.

a Some samples have a Mn or Cu concentration lower than the respective detection limit, and half of the detection limit was used as their values for ease of statistics. Data are presented as mean ± standard deviation.

b Guidelines are given for comparison according to the national standards for drinking water quality [13].

c The significance of difference regarding different sample-classification criteria in the first column is described using *p* values.

Water samples from the office building have a high Zn content (0.40 ± 0.24 mg L^−1^, *p* < 0.001 in ANOVA). Particularly, the Zn concentration in samples “O_19_EtOH” and “O_21_fre” reaches up to 0.87–0.95 mg L^−1^, which is close to the guideline given in the standards (1.0 mg L^−1^). Tap waters sampled from the higher storeys (19th–34th) show a higher Zn content (0.50 ± 0.22 mg L^−1^) compared to the lower storeys (2nd–7th, 0.22 ± 0.16 mg L^−1^) with statistical significance (*p* = 0.015). The maximal HPC (98 CFU mL^−1^) was determined in sample “O_2_fre”, which almost reaches up to the guideline standard (100 CFU mL^−1^). In terms of different water consumption levels, water sampled from the frequently-used taps (10–14 t month^−1^) has a higher sulfate content (18.7 ± 1.0 mg L^−1^, *p* = 0.015) and a lower Fe concentration (0.024 ± 0.006 mg L^−1^, *p* = 0.022) compared to the water samples from the less-used taps (1–5 t month^−1^). An estimation of water storage time further shows that the longer HRT within SWSSs leads to Fe dissolution, with a correlation coefficient 0.79 (*p* < 0.001).

### 3.2 Bacterial community composition

The α-diversity of the bacterial community is higher in the office samples than in the residential samples (Table S4, *p* < 0.001 in ANOVA). Samples from Tank 2 (located on the mechanical floor of the office building), namely “T2_fre” and “T2_UV”, are also characterized by high bacterial diversity. Besides, the high bacterial diversity was also determined, through a statistical analysis based on Tables S1 and S4, in the samples from the less-used taps (3–5 t month^−1^) compared to the frequently-used taps (10–26 t month^−1^) in the residential building (*p* = 0.04), and lower storeys (2nd–7th) compared to higher storeys (19th–34th) in the office building (*p* < 0.001). In contrast, no significant difference in bacterial diversity could be associated with tap disinfection, tap locations, and post-flushing.

**Fig. 2:**
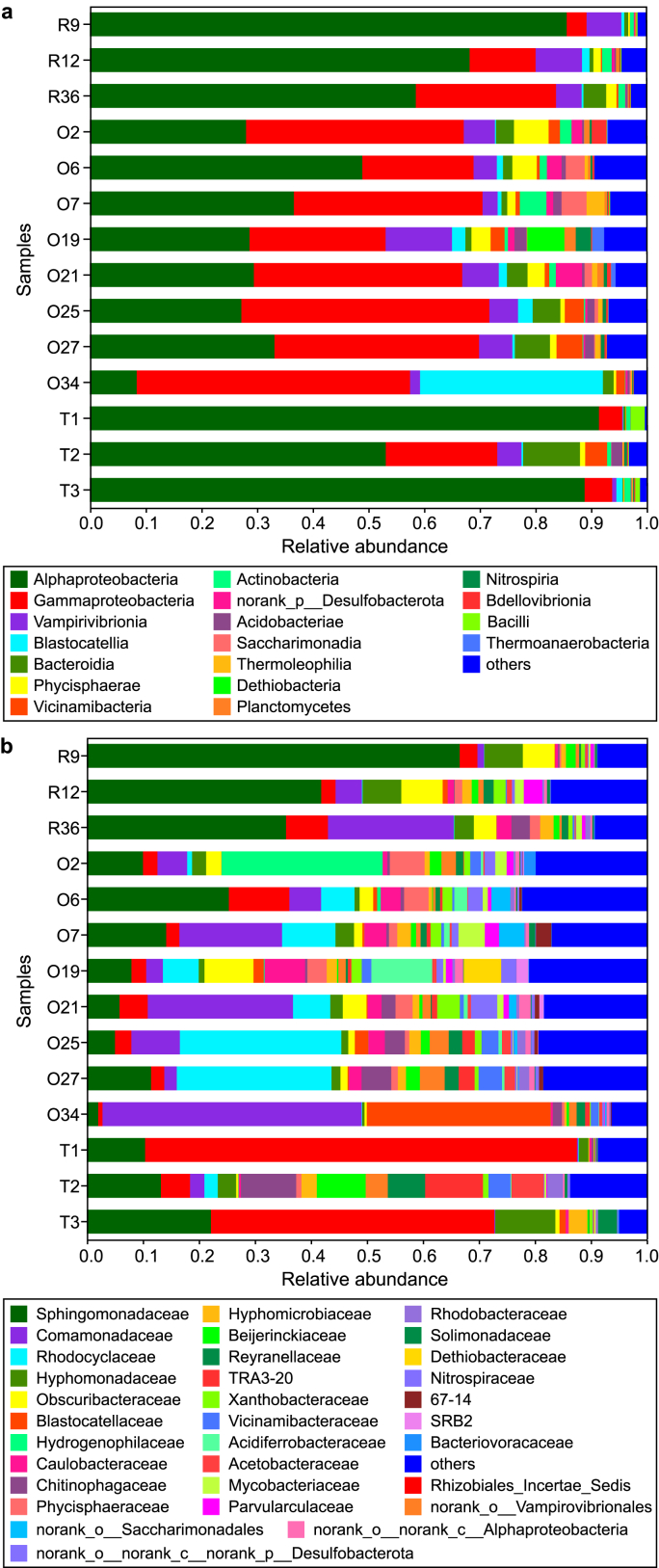
Bacterial community composition at class (**a**) and family (**b**) levels. The average was calculated for each floor. Taxons constituting <2% of the community were classified as “others”.

Proteobacteria is dominant in all samples, accounting for 51.0–99.4% of the bacterial community, whereas the proportion of classes varies in different samples (Fig. 2a). Fig. S1 shows a heatmap of the top 30 dominant ASVs, of which the taxonomy information is given in Table S5. With a relative abundance of 41.6–96.3%, α-Proteobacteria is the dominant class in the samples from residences and water tanks, mainly including the genera *Phreatobacter*, *Porphyrobacter*, *Blastomonas*, *Sphingomonas*, and the family Hyphomonadaceae (Fig. S1 and Table S5). In contrast, most of the office samples are dominated by γ-Proteobacteria (28.7–49.1%), including the genera *Aquabacterium*, *Methyloversatilis*, *Hydrogenophaga*, and the family Rhodocyclaceae, with the average abundance of α-Proteobacteria decreasing to 27.1%.

As for the water tank samples, the bacterial community composition bars (Fig. 2b) and the heatmap (Fig. S1) demonstrate the higher abundance of the genera *Sediminibacterium*, *Reyranella*, *Bosea*, and the family TRA3-20 in the samples from Tank2, which are almost negligible in other samples. *Phreatobacter*, belonging to the order Rhizobiales, accounts for 77.1% in “T1_fre” and 50.5% in “T3_UV”, while its relative abundance is only <1–16.8% in the remaining samples.

### 3.3 β-Diversity comparing bacterial community similarity

**Fig. 3:**
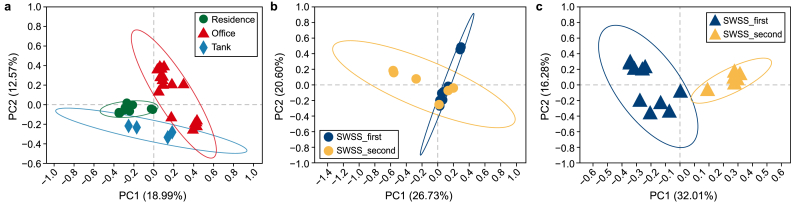
Principal coordinate analysis (PCoA) plots for all samples grouped based on the functional area (**a**), residential samples (**b**), and office samples grouped based on the water supply strategy (**c**). The ellipses represent the variation of each group at a confidence level of 95%.

Through data dimensionality reduction based on ASV abundance, PCoA illustrates the similarities or differences among bacterial communities. Samples from the same functional area cluster on the PCoA plot (Fig. 3a), indicating inner-group similarity and inter-group heterogeneity of bacterial community composition, with an ANOSIM result of *R* = 0.621 and *p* = 0.001. Panels b and c in Fig. 3 aim to further explore the sample similarity in residential and office sub-groups separately. In this case, the water supply strategy also seems to influence the bacterial community structure, with an ANOSIM result of *R* = 0.216, *p* = 0.047 for residence and *R* = 0.793, *p* = 0.001 for office.

### 3.4 Microbial growth potential and occurrence of *Mycobacterium*

**Fig. 4:**
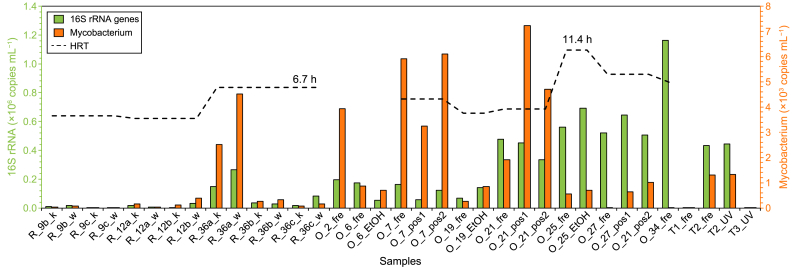
Enumeration of 16S rRNA genes and the genus *Mycobacterium*.

The numbers of 16S rRNA genes are significantly higher (*p* = 0.02 in ANOVA) in the tap water samples from the office building (including Tank2) than in the residential building (Fig. 4). This difference could be caused by metal leaching, as the correlation coefficient *R*^2^ is 0.437 (*p* = 0.04) between the 16S rRNA gene copy number and Zn concentration. Particularly, higher numbers were observed in the high-storey samples (mainly “SWSS-second”), with the maximum determined on the top floor (sample “O_34_fre”) of the office building. Similarly, the numbers of 16S rRNA genes for the residential building are generally 1–2 orders of magnitudes higher in the water samples from the 36th floor than the 9th and 12th floors. The ANOVA results show that the water supply strategy significantly affected the numbers of 16S rRNA, with *p* = 0.027 for the residential building and *p* = 0.001 for the office building. The HRT estimation result is also displayed in Fig. 4, which exhibits a significant correlation with the 16S rRNA gene copy number (*R*^2^ = 0.63, *p* < 0.01).

Generally, the genus *Mycobacterium*, long-recognized as prevalent in drinking waters and associated with opportunistic pathogens [[3], [4], [5]], accounts for 0–2% of the bacterial community, whereas its abundance reaches 4.0% in “R_12b_k”, 2.0% in “O_2_fre”, and 3.6–5.7% in “O_7” samples. From a quantitative perspective, qPCR results demonstrate the occurrence of *Mycobacterium* in “R_36a”, “O_2”, “O_7”, and “O_21” samples (Fig. 4).

### 3.5 Correlation between bacterial community and water quality

**Fig. 5:**
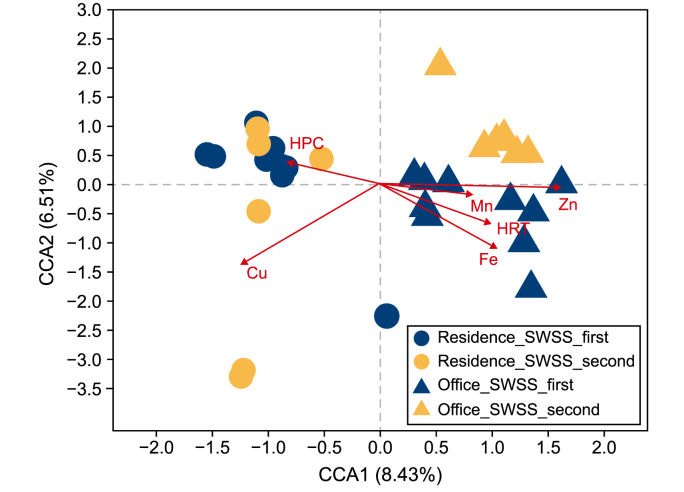
Canonical correspondence analysis (CCA) shows the correlation between bacterial community and water physiochemical parameters.

The effects of functional areas and water supply strategies on the bacterial community are also evident under constrained ordination with CCA (Fig. 5). These effects are significantly associated with the concentrations of Zn and Cu (*p* = 0.001) and the HRT (*p* = 0.006) in tap water. Cu is the primary factor driving bacterial community shift in the residential water, while Zn plays a vital role in shaping the bacterial community of samples from the office. Mainly determined by plumbing topology and meanwhile influenced by household water consumption in SWSSs, HRT is a comprehensive factor accounting for the shift in bacterial community profiles with different floors and water supply strategies.

## 4 Discussion

### 4.1 Shifts in the bacterial community in SWSSs

A broad geographical survey indicates that the distribution process produces significant selective effects on the tap water microbiome, presumably enforced by chlorination and darkness [29,30]. It contributes to the comparative simplicity and relative homogeneity of microbiology regardless of geographical location, land use, and source water type [29]. Studies concerning a full-scale source-to-tap water distribution network also indicate that the rare taxa displayed dynamicity while the dominant taxa remained stable [25]. Nevertheless, this study demonstrates that even with identical source water, treatment strategy, and distribution process, SWSSs play an important role in shaping the bacterial community in tap water at the distal ends. Bacterial populations exhibit distinct sensitivity to environmental factors, driving the succession and diversification of microbiomes during water distribution.

#### 4.1.1 Effects of functional areas and plumbing materials

Building-specific microbial communities in tap water have been reported previously [10], which resulted from a combined effect of source water quality and plumbing features. This study further demonstrates that tap water originating from the same underground water tank has distinct microbiomes for different buildings (Fig. 3a), highlighting the significant role of SWSSs. These shifts in the microbial community are mainly associated with water chemistry (Fig. 5), implying the effect of plumbing materials in SWSSs.

The high Cu concentration in tap water from the residential building (Table 1) could result from the leaching of brass taps and fittings commonly used in household plumbing for aesthetics [8]. Generally, bacterial diversity decreases after chlorination and increases along the distribution pipeline [30], which could be inferred to happen during secondary water supply. However, abiotic factors such as Cu stress could kill some sensitive bacterial populations and cause a shift in bacterial community [12,31,32]. It elucidates the significantly lower bacterial diversity in tap water from the residential building compared to the office building (Table S4).

Entrainment of zinc component from outside galvanized layers, which could be oxidized and released, especially with increasing temperature [33], is responsible for the high Zn concentration in office tap water samples (Table 1), as all the horizontal pipes in this building are made of GPLS. By contrast, the inhibiting effect on bacterial diversity produced by Zn stress is much weaker than Cu stress (Table S4 and Fig. 4), which has also been reported in a previous study [31]. Nevertheless, exposure to Zn-abundant water also produces a selective effect on metal-sensitive species. It alters bacterial community, e.g., decreasing the abundance of *Sphingomonas* (Fig. S1 and Table S5), which has been classified as a metal-intolerant taxon [34].

Compared to taps in washrooms and kitchens, taps equipped in water tanks reserved for routine inspection and maintenance are seldom used. The prolonged stagnation of water near taps is liable to the formation of loose deposits and unstable corrosion scales on the inner plumbing surface [[35], [36], [37], [38], [39]], which could be potentially released during hydraulic peaks such as tap opening, leading to the high Fe concentration in the samples from water tanks (Table 1). Besides, green rusts, a basic kind of corrosion product containing Fe(II), Fe(III), and Cl^−^/SO_4_^2−^/CO_3_^2−^, are unstable in the distribution systems [40]. The release of corrosion scales containing green rusts in these seldomly-used taps may account for the high chloride in tank water samples, with an increasing TDS. These distinct pipe deposits and water physicochemical properties contribute to the particularity of water microbiomes (Fig. 2b).

#### 4.1.2 Water supply strategy-associated variations

Despite consistent pipe fitting materials and a stable intra-building environment, the tap water microbiome can vary based on water supply strategies (Fig. 3b and c). An increasing number of total bacteria (Fig. 4) combined with the alteration of the bacterial community (Fig. 2b) is identified in the “SWSS-second” samples compared to “SWSS-first”. The estimated HRT further demonstrates the increased potential of microbial regrowth (Fig. 4) and shift in bacterial community profile (Fig. 5) with prolonged storage and transportation. Served directly by the mains, tap water from the 2nd floor of the office building has a higher number of HPC (Table 1) compared to the others supplied by SWSSs with ultraviolet disinfection. Correspondingly, the bacterial community composition, dominated by *Hydrogenophilaceae*, is distinctive from the remaining samples (Fig. 2b). This result contradicts the previous finding [14], which shows the deterioration of physiochemical and microbiological water quality in SWSSs compared to “direct supply” systems. The discrepancy is due to secondary disinfection in our studied case, which is absent in the area investigated by Zhang et al. [14]. It implies that water storage in SWSSs significantly changes the microbiome at distal ends, and secondary disinfection is necessary to protect the water quality stability in SWSSs.

#### 4.1.3 Contributions of water consumption levels

The frequency of tap utilization could, directly and indirectly, influence the bacterial community in tap water. On the one hand, the prolonged stagnation period potentially allows for microbial regrowth, thus causing a shift in community profiles [6,12]. On the other hand, biofilms, deposit layers, and metal corrosion scales have been formed inside the pipes and plumbing surfaces during years of service, which are significantly influenced by the hydraulic conditions and water age [[37], [38], [39]]. The increased retention time in the pipe ends of less-used taps tends to form loose deposits, which have a high potential of resuspension during hydraulic peaks [2,35,36,41]. This is consistent with the high Fe concentration in the water sampled from the less-used taps (Table 1). The particularity in pipe deposits and water chemistry contributes to the variation in bacterial community.

Besides, hydraulic regimes determine the selection of seed bacteria from bulk water that attach and multiply on plumbing surfaces [42,43], affecting the bacterial community profiles in tap water. In this study, the significant influence of SWSS plumbing features and water supply strategy might mask the effect of tap utilization frequency on the water microbiome, making the water consumption-associated difference in bacterial communities comparatively difficult to distinguish. Nevertheless, lower bacterial diversity was determined in the samples from the frequently-used taps, which could have resulted from the selection and elimination effects during hydraulic peaks.

#### 4.1.4 Other influencing factors in SWSSs

This study found that post-flushing, tap disinfection, and tap location (in the kitchen or washroom for residence) exert only marginal effects on tap water microbiomes. Generally, the “first-draw” sample represents the water that has been stagnant in the supply lines overnight and, in comparison, the post-flushing sample indicates water from the standpipes, water tanks, or water mains depending on the water supply strategies and the flushing volumes. Compared with post-flushing samples, significant shifts in bacterial community [10,14], increase of bacterial activity and opportunistic pathogens [11,14], and deterioration of metallic water quality [8,14] have been determined after overnight stagnation. However, this study observed an indistinctive difference in water quality and bacterial community between the “first-draw” and post-flushing samples. As the pipe diameter of the supply line is 25–30 mm, the 2-L “first-draw” sample represents water stored overnight in the adjacent 3–4 m of the supply line. In contrast, after 10- and 18-L flushing, the samples can be considered water from the standpipes and tanks (Fig. 1). The stable water quality could be ascribed to the effectiveness of secondary disinfection and storage time control, as the estimated HRT is 6–12 h in average. Nevertheless, further investigation remains to investigate how the effect would change with the system aging and the pattern after longer stagnation (e.g., long holidays).

### 4.2 Practical implications and research prospects

Although significant shifts in bacterial community were determined during secondary water supply, these variations did not imply certain hygienic issues. As declared in previous publications [25,44], biological stability and microbiologically safe water concepts need further investigation. A failed amplification of the typical opportunistic pathogen genes and an indistinctive difference in “first-draw” samples indicate the satisfactory microbiological quality of water in this case. A higher number of total bacteria reflected by the 16S rRNA genes implies a higher potential of microbial regrowth in the office SWSSs (Fig. 4). However, this issue may not warrant excessive concern, as the tap water in office settings is primarily used for handwashing, rather than drinking or cooking.

It must be pointed out that although following certain standards, the exact design of SWSSs varies in different high-rise buildings, associated with the height, function, and structure of buildings. Nevertheless, the architectural complex selected in this study consists of different functional areas and involves various water supply strategies. Meanwhile, the sampling campaign considers many factors, including storey, frequency of tap utilization, tap location, tap disinfection, and post-flushing. Therefore, the findings of intra-SWSSs variations provide important insights into maintaining water quality stability during secondary water supply. For example, a higher potential of microbial regrowth in office SWSSs indicates that appropriate pipe and fittings materials can be selected to protect the stability of microbiological water quality, which is applicable both for SWSSs and premise plumbing. Secondary disinfection is important in protecting microbiological water quality in high-rise buildings, while the stroeys supplied directly by mains should be paid more attention. In addition, the periodical flushing of dead ends and effective regulation of HRT can be simultaneously adopted to populate a desirable microbial community to compete in ecological niches with the undesirable micro-organisms associated with opportunistic pathogens [4,7]. Long-term observation of microbial water quality will be performed, as conducted in pipe distribution systems [45,46], to include the temporal variations and seasonal dynamics, based on which a pre-set quantitative (cell number) and qualitative (bacterial community) threshold could be established for developing an early-warning methodology.

## 5 Conclusions

A thorough investigation into the microbiological quality of water demonstrates that SWSSs significantly influence tap water microbiomes at the distal ends. The materials of pipe fittings in various buildings, various water supply strategies on different floors, and changing water flow conditions near the taps produce a combined effect on water chemistry and storage time, subsequently determining the bacterial community profiles in tap water. Accordingly, practical technologies, such as optimization of SWSS design, appropriate selection of pipe fittings materials, and effective regulation of HRT, are developed to protect the microbiological water quality at consumers’ taps. Future studies will be conducted to identify the seasonal variations in the tap water microbiome to establish an early-warning methodology of water quality stability in SWSSs.

## CRediT authorship contribution statement

**Manjie Li:** Conceptualization, Methodology, Investigation, Formal Analysis, Writing Original Draft, Visualization. **Zhaowei Liu:** Validation, Writing - Review & Editing. **Yongcan Chen:** Resources, Supervision.

## Declaration of competing interest

The authors declare that they have no known competing financial interests or personal relationships that could have appeared to influence the work reported in this paper.
