# Supplementary material for: Tap water microbiome shifts in secondary water supply for high-rise buildings
Source: Environ Sci Ecotechnol. 2024 Mar 16;20:100413. doi: 10.1016/j.ese.2024.100413 (PMC10997949; doi:10.1016/j.ese.2024.100413)
Supplement: Multimedia component 1 [file mmc1.pdf]

Supplementary Material

Tap Water Microbiome Shifts in Secondary Water Supply for High-rise Buildings

Manjie Li[a,b], Zhaowei Liu[b,*], and Yongcan Chen[b]

[a] Shenzhen International Graduate School, Tsinghua University, Shenzhen 518055, P.R. China

[b] State Key Laboratory Hydroscience and Engineering, Tsinghua University, Beijing 100084, P.R. China

[*] Corresponding Author.

Zhaowei Liu, Tel: +86 10 62770207, E-mail: liuzhw@tsinghua.edu.cn

9 Pages: 5 Tables, 1 Figure

Table S1. Sample information.

Sample ID	Functional areas	Storey	Water supply	Water consumption[v]	Remarks	Sample ID	Functional areas	Storey	Water supply	Water consumption	Remarks
R_9a_k[i]	Residence	9th	SWSS-first[iv]	10 t/month	Failed in sampling	O_2_fre[ii]	Office	2nd	Directly served by the mains	-[vi]	-
R_9a_w	Residence	9th	SWSS-first			O_6_fre	Office	6th	SWSS-first	1 t/month	-
R_9b_k	Residence	9th	SWSS-first	10 t/month	-	O_6_EtOH	Office	6th	SWSS-first		-
R_9b_w	Residence	9th	SWSS-first		-	O_7_fre	Office	7th	SWSS-first	10 t/month	-
R_9c_k	Residence	9th	SWSS-first	3 t/month	-	O_7_pos1	Office	7th	SWSS-first		-
R_9c_w	Residence	9th	SWSS-first		-	O_7_pos2	Office	7th	SWSS-first		-
R_12a_k	Residence	12th	SWSS-first	20 t/month	-	O_19_fre	Office	19th	SWSS-first	5 t/month	-
R_12a_w	Residence	12th	SWSS-first		-	O_19_EtOH	Office	19th	SWSS-first		-
R_12b_k	Residence	12th	SWSS-first	5 t/month	-	O_21_fre	Office	21st	SWSS-first	3 t/month	-
R_12b_w	Residence	12th	SWSS-first		-	O_21_pos1	Office	21st	SWSS-first		-
R_12c_k	Residence	12th	SWSS-first	3 t/month	Failed in sampling	O_21_pos2	Office	21st	SWSS-first		-
R_12c_w	Residence	12th	SWSS-first			O_25_fre	Office	25th	SWSS-second	3 t/month	-
R_36a_k	Residence	36th	SWSS-second	11 t/month	-	O_25_EtOH	Office	25th	SWSS-second		-
R_36a_w	Residence	36th	SWSS-second		-	O_27_fre	Office	27th	SWSS-second	14 t/month	-
R_36b_k	Residence	36th	SWSS-second	3 t/month	-	O_27_pos1	Office	27th	SWSS-second		-
R_36b_w	Residence	36th	SWSS-second		-	O_27_pos2	Office	27th	SWSS-second		-
R_36c_k	Residence	36th	SWSS-second	26 t/month	-	O_34_fre	Office	34th	SWSS-second	13 t/month	-
R_36c_w	Residence	36th	SWSS-second		-	T1_fre[iii]	Tank1	Underground	-	-	-
T3_fre	Tank3	30th	-	-	Failed in sampling	T1_UV	Tank1	Underground	-	-	Failed in DNA amplification
T3_UV	Tank3	30th	-	-	-	T2_fre	Tank2	23rd	-	-	-
						T2_UV	Tank2	23rd	-	-	-

[i]For the residential building, three apartments were sampled on each floor and marked with “a”, “b”, and “c”, respectively. For each apartment, both the kitchen tap water and washroom tap water were sampled and marked with “k” and “w”, respectively.

[ii]For the office building, the “first-draw” sample was taken on the eight selected floors, marked with “fre”. On the 6th, 19th and 25th floors, an extra sample was taken following tap disinfection with 75% ethanol, marked with “EtOH”. On the 7th, 21st and 27th floors, two extra post-flushing samples were taken after 10-L and 18-L flushing and marked with “pos1” and “pos2”, respectively.

[iii]For the water tanks, water was sampled from the underground tank, the tanks on the 23rd floor of the office building, and the tank on the 30th floor of the residential building and marked with “T1”, “T2”, and “T3”, respectively. Samples were taken before and after ultraviolet disinfection and marked with “fre” and “UV”, respectively.

[iv]The consumers from the 3rd floor to the mechanical floor (the 23rd floor for the office building and the 30th floor for the residential building) are served with the water from the underground tank, marked with “SWSS-first”. The rest above are served with the water stored in the tanks on the mechanical floor, marked with “SWSS-second” (Figure 1).

[v]Water consumption is recorded monthly for each apartment in the residential building and for each floor in the office building. Data of water consumption in summer were given.

[vi]Water consumption has not been monitored on the 2nd floor which is the lobby of the office building.

Table S2. Barcode information.

Samples	FBarcode	RBarcode	Samples	FBarcode	RBarcode
R_9b_k	AGCATG	TTGACG	O_2_fre	TCTGTC	TGCAAG
R_9b_w	GTGAAC	CTGTTC	O_6_fre	TTAGCC	ACTGCT
R_9c_k	CGCATA	GTACTC	O_6_EtOH	ACGAAG	TACTCG
R_9c_w	TGTGCA	CCGTAA	O_7_fre	GTCTGT	CTCTAG
R_12a_k	AGTTCC	TGAATG	O_7_pos1	CAGTGT	GAGTGT
R_12a_w	GTACTT	CCAGCT	O_7_pos2	TGTGTC	ATCTCC
R_12b_k	CAGATC	GTGAAA	O_19_fre	GTACCT	CTACCA
R_12b_w	TAATCG	ACTTGA	O_19_EtOH	CGAGAT	GCTTCA
R_36a_k	ATCACG	TACAGC	O_21_fre	AGAGCT	TGCCAA
R_36a_w	GAGATA	CTAGCT	O_21_pos1	GCACAA	CCTTCT
R_36b_k	CGCGGT	GAGTGG	O_21_pos2	CATTCG	GACTTC
R_36b_w	TCGGCA	AGTCAA	O_25_fre	TAACGC	CGTAAC
R_36c_k	ATCGTA	TCTACC	O_25_EtOH	ACTCAC	TGACAC
R_36c_w	GGTTGT	CGATGT	O_27_fre	TCCACA	ATTGCG
T1_fre	CCACAA	GCCAAT	O_27_pos1	AGATCC	TCGTTG
T2_fre	ACAGTG	TTCAGA	O_27_pos2	GCAGTT	CACCAA
T2_UV	GTTGAA	CATCGT	O_34_fre	CCTATG	GTCATG
T3_UV	CGTACG	GCCGCG			

Table S3. Primers, probes, and amplification conditions used in quantitative polymerase chain reaction (Li et al., 2018; Wang et al., 2012).

Targeted organisms	Targeted genes	Sequences (5'-3')	Annealing temperature	Reference
**Total bacteria**	16S rRNA	1368F: CGGTGAATACGTTCYCGG<br>1492R: GGWTACCTTGTTACGACTT	55 °C	(Suzuki et al., 2000)
***Mycobacterium* spp.**	16S rRNA	110F: CCTGGGAAACTGGGTCTAAT<br>I571R: CGCACGCTCACAGTTA<br>H19R: FAM-TTTCACGAACAACGCGACAAACT	56 °C	(Radomski et al., 2010)
***Legionella* spp.**	23S rRNA	Leg23SF: CCCATGAAGCCCGTTGAA<br>Leg23SR: ACAATCAGCCAATTAGTACGAGTTAC<br>Probe: HEXTCCACACCTCGCCTATCAACGTCGTAGT	58.5 °C	(Nazarian et al., 2008)
L. pneumophila	*mip*	LmipF: AAAGGCATGCAAGACGCTATG<br>LmipR: GAAACTTGTTAAGAACGTCTTTCATTTG<br>Probe: FAM-TGGCGCTCAATTGGCTTTAACCGA	60 °C	(Nazarian et al., 2008)
M. avium	16S rRNA	MycavF: AGAGTTTGATCCTGGCTCAG<br>MycavR: ACCAGAAGACATGCGTCTTG	64 °C	(Wilton and Cousins, 1992)
***Acanthamoeba* spp.**	18S rRNA	TaqAcF1: CGACCAGCGATTAGGAGACG<br>TaqAcR1: CCGACGCCAAGGACGAC<br>Probe: FAM-TGAATACAAAACACCACCATCGGCGC	60 °C	(Rivière et al., 2006)
V. vermiformis	18S rRNA	Hv1227F: TTACGAGGTCAGGACACTGT<br>Hv1728R: GACCATCCGGAGTTCTCG	60 °C	(Kuiper et al., 2006)

Table S4. Alpha-diversity indices of the bacterial communities.

Samples	Chao1/Ace	Shannon	Simpson
R_9b_k	219	1.61	0.507
R_9b_w	213	1.97	0.355
R_9c_k	286	2.97	0.142
R_9c_w	439	3.99	0.055
R_12a_k	320	4.11	0.033
R_12a_w	251	3.21	0.101
R_12b_k	369	3.76	0.067
R_12b_w	467	4.41	0.030
R_36a_k	276	3.51	0.077
R_36a_w	231	3.51	0.097
R_36b_k	296	3.32	0.087
R_36b_w	582	4.43	0.036
R_36c_k	310	2.70	0.173
R_36c_w	91	1.94	0.269
O_2_fre	656	4.33	0.089
O_6_fre	541	4.08	0.059
O_6_EtOH	738	4.82	0.029
O_7_fre	741	4.92	0.020
O_7_pos1	616	4.44	0.033
O_7_pos2	639	4.60	0.031
O_19_fre	499	4.46	0.034
O_19_EtOH	532	4.75	0.019
O_21_fre	517	4.46	0.040
O_21_pos1	512	4.46	0.031
O_21_pos2	484	4.32	0.039
O_25_fre	607	4.39	0.048
O_25_EtOH	577	4.40	0.043
O_27_fre	532	3.72	0.134
O_27_pos1	496	4.07	0.084
O_27_pos2	531	4.35	0.041
O_34_fre	349	2.12	0.312
T1_fre	236	1.32	0.600
T2_fre	524	4.18	0.035
T2_UV	348	3.53	0.062
T3_UV	283	2.43	0.277

Table S5. Taxonomy information of the top-30 dominant ASVs.

ASV ID	Taxonomy				
	Phylum	Class	Order	Family	Genus
ASV4	Proteobacteria	Alphaproteobacteria	Rhizobiales	Rhizobiales_Incertae_Sedis	Phreatobacter
ASV17	Proteobacteria	Alphaproteobacteria	Sphingomonadales	Sphingomonadaceae	Porphyrobacter
ASV2	Proteobacteria	Alphaproteobacteria	Sphingomonadales	Sphingomonadaceae	Blastomonas
ASV3	Proteobacteria	Alphaproteobacteria	Sphingomonadales	Sphingomonadaceae	Sphingomonas
ASV5	Proteobacteria	Alphaproteobacteria	Caulobacterales	Hyphomonadaceae	
ASV173	Proteobacteria	Gammaproteobacteria	Burkholderiales	Comamonadaceae	Aquabacterium
ASV257	Proteobacteria	Gammaproteobacteria	Burkholderiales	Rhodocyclaceae	Methyloversatilis
ASV14	Proteobacteria	Gammaproteobacteria	Burkholderiales	Comamonadaceae	Aquabacterium
ASV443	Proteobacteria	Gammaproteobacteria	Burkholderiales	Comamonadaceae	Aquabacterium
ASV59	Bacteroidota	Bacteroidia	Chitinophagales	Chitinophagaceae	Sediminibacterium
ASV1	Cyanobacteria	Vampirivibrionia	Obscuribacterales	Obscuribacteraceae	
ASV10	Proteobacteria	Alphaproteobacteria	Sphingomonadales	Sphingomonadaceae	
ASV54	Planctomycetota	Phycisphaerae	Phycisphaerales	Phycisphaeraceae	SM1A02
ASV270	Proteobacteria	Gammaproteobacteria	Burkholderiales	Comamonadaceae	Zhizhongheella
ASV1241	Proteobacteria	Gammaproteobacteria	Burkholderiales	Rhodocyclaceae	Methyloversatilis
ASV456	Proteobacteria	Gammaproteobacteria	Burkholderiales	Rhodocyclaceae	
ASV824	Proteobacteria	Alphaproteobacteria	Sphingomonadales	Sphingomonadaceae	Sphingopyxis
ASV20	Proteobacteria	Alphaproteobacteria	Sphingomonadales	Sphingomonadaceae	Sphingomonas
ASV900	Proteobacteria	Gammaproteobacteria	Burkholderiales	TRA3-20	
ASV440	Proteobacteria	Gammaproteobacteria	Burkholderiales	Comamonadaceae	Hydrogenophaga
ASV290	Acidobacteriota	Blastocatellia	Blastocatellales	Blastocatellaceae	
ASV307	Proteobacteria	Gammaproteobacteria	Burkholderiales	Hydrogenophilaceae	Thiobacillus
ASV1713	Proteobacteria	Alphaproteobacteria	Sphingomonadales	Sphingomonadaceae	Novosphingobium
ASV901	Proteobacteria	Alphaproteobacteria	Rhizobiales	Beijerinckiaceae	Bosea
ASV11	Proteobacteria	Alphaproteobacteria	Sphingomonadales	Sphingomonadaceae	Sphingomonas
ASV902	Proteobacteria	Alphaproteobacteria	Reyranellales	Reyranellaceae	Reyranella
ASV441	Proteobacteria	Gammaproteobacteria	Burkholderiales	Rhodocyclaceae	Methyloversatilis
ASV448	Patescibacteria	Saccharimonadia	Saccharimonadales		
ASV905	Acidobacteriota	Vicinamibacteria	Vicinamibacterales	Vicinamibacteraceae	
ASV229	Proteobacteria	Alphaproteobacteria	Rhizobiales	Rhizobiales_Incertae_Sedis	Phreatobacter

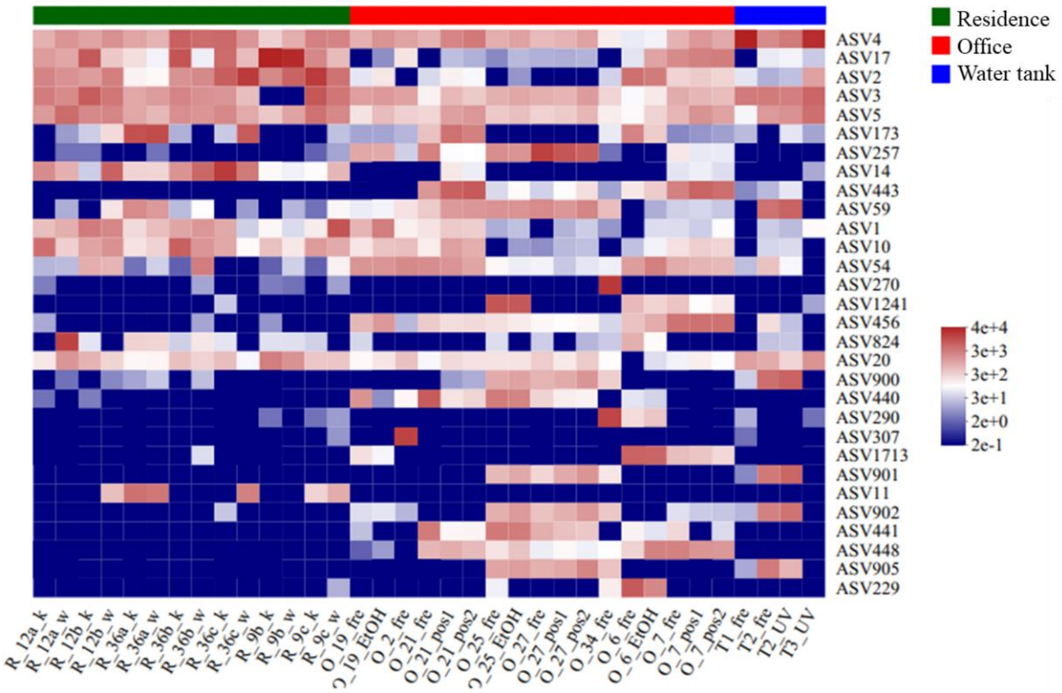

Figure S1. Heatmap showing the top-30 abundant ASVs in all samples, with the taxonomy information given in Table S5.

References

Kuiper, M.W., Valster, R.M., Wullings, B.A., Boonstra, H., Smidt, H., van der Kooij, D., 2006. Quantitative Detection of the Free-Living Amoeba *Hartmannella vermiformis* in Surface Water by Using Real-Time PCR. Appl. Environ. Microbiol. 72, 5750–5756. https://doi.org/10.1128/AEM.00085-06

Li, H., Li, S., Tang, W., Yang, Y., Zhao, J., Xia, S., Zhang, W., Wang, H., 2018. Influence of secondary water supply systems on microbial community structure and opportunistic pathogen gene markers. Water Res. 136, 160–168. https://doi.org/10.1016/j.watres.2018.02.031

Nazarian, E.J., Bopp, D.J., Saylors, A., Limberger, R.J., Musser, K.A., 2008. Design and implementation of a protocol for the detection of Legionella in clinical and environmental samples. Diagn. Microbiol. Infect. Dis. 62, 125–132. https://doi.org/10.1016/j.diagmicrobio.2008.05.004

Radomski, N., Lucas, F.S., Moilleron, R., Cambau, E., Haenn, S., Moulin, L., 2010. Development of a Real-Time qPCR Method for Detection and Enumeration of *Mycobacterium* spp. in Surface Water. Appl. Environ. Microbiol. 76, 7348–7351. https://doi.org/10.1128/AEM.00942-10

Rivière, D., Szczebara, F.M., Berjeaud, J.-M., Frère, J., Héchard, Y., 2006. Development of a real-time PCR assay for quantification of Acanthamoeba trophozoites and cysts. J. Microbiol. Methods 64, 78–83. https://doi.org/10.1016/j.mimet.2005.04.008

Suzuki, M.T., Taylor, L.T., DeLong, E.F., 2000. Quantitative Analysis of Small-Subunit rRNA Genes in Mixed Microbial Populations via 5′-Nuclease Assays. Appl. Environ. Microbiol. 66, 4605–4614. https://doi.org/10.1128/AEM.66.11.4605-4614.2000

Wang, H., Edwards, M., Falkinham, J.O., Pruden, A., 2012. Molecular Survey of the Occurrence of Legionella spp., Mycobacterium spp., Pseudomonas aeruginosa, and Amoeba Hosts in Two Chloraminated Drinking Water Distribution Systems. Appl. Environ. Microbiol. 78, 6285–6294. https://doi.org/10.1128/AEM.01492-12

Wilton, S., Cousins, D., 1992. Detection and identification of multiple mycobacterial pathogens by DNA amplification in a single tube. PCR Methods Appl. 1, 269–273.
